# Influence of light conditions (colour temperature and illuminance) on the evaluation of root translucency for the application of Lamendin’s age-at-death estimation technique

**DOI:** 10.1007/s00414-022-02902-1

**Published:** 2022-10-20

**Authors:** Joan Viciano, Iuri Icaro, Carmen Tanga, Domenico Tripodi

**Affiliations:** 1 Department of Medicine and Aging Sciences, ‘G. d’Annunzio’ University of Chieti-Pescara, Chieti, Italy; 2 Department of Legal Medicine, Toxicology and Physical Anthropology, University of Granada, Granada, Spain; 3 Department of Medical and Oral Sciences and Biotechnologies, ‘G. d’Annunzio’ University of Chieti-Pescara, Chieti, Italy

**Keywords:** Forensic anthropology, Lamendin’s technique, Dental age estimation, Root translucency, Light conditions

## Abstract

Estimation of age-at-death represents a central focus in forensic human identification, as it is a key parameter used in the identification of unidentified bodies. In 1992, Lamendin et al. published a simple technique for estimating the age-at-death of adult skeletal remains based on two dental criteria: the gingival regression and the extent of dentine translucency. Although Lamendin’s technique is widely used in forensic contexts and the evaluation of root translucency is a key element in the technique, the light conditions for measuring this parameter have not been adequately established. The aim of the present study is to analyse the influence of colour temperature and illuminance level of a LED light source when root translucency is evaluated to optimize the use of Lamendin’s technique for age-at-death estimation. The results describe how light settings may affect the visual perception of root translucency by different examiners and, therefore, affect the accuracy of the age-at-death estimation methods and techniques based on this parameter.

## Introduction

The estimation of age-at-death from skeletal remains is one of the main challenges for forensic anthropologists and represents a key parameter used to identify unknown individuals [1]. In 1992, Lamendin et al. [2] published a simple technique for estimating the age-at-death of adult skeletal remains based on two dental criteria: the gingival regression and the extent of dentine translucency. Both variables were measured on the labial surface of single-rooted teeth and expressed in relation to the total root length. Lamendin’s technique and its derived modifications (e.g. [3, 4]) attracted considerable attention for forensic purposes because its accuracy, speed and simplicity due to its application was based on simple observations from intact teeth, and it did not require prior training or special equipment, only requiring a caliper and an adequate light source to record the root translucency.

Since the initial development of Lamendin’s technique, several types of variability conditions were evaluated to optimize the use of this technique, such as (i) target teeth to which this technique is applied (i.e. differences between the diverse classes of single-rooted teeth (incisors, canines, premolars) or between maxillary and mandibular teeth); (ii) dental surface analysed (i.e. differences between labial/buccal or palatal/lingual tooth surfaces); (iii) biological profile of the analysed individual (i.e. differences between the sexes or between different populations); (iv) statistical strength for the development of the methodology; (v) environmental conditions (i.e. taphonomic impact and postmortem interval in dental tissue diagenesis); (vi) discrepancies in the application of the technique by different examiners (i.e. repeatability and reproducibility of the collected measurements) and (vii) other factors other than age that influence gingival regression and root translucency (e.g. dental pathologies, direct (on sectioned teeth) or indirect (on intact teeth) techniques) (see Parra et al. [5] for more details on these variability conditions for the application of Lamendin’s technique).

Although Lamendin’s technique is widely used in forensic contexts and the evaluation of root translucency is a key element in the technique, light conditions for measuring this parameter have not been adequately established. Light settings used for measuring root translucency have not always been reported. Some authors only reported that root translucency was measured by placing the tooth against a bright light source, such as a light box or natural light, but did not provide further details (e.g. [3, 6–10]). Other authors provided more details of the light source such as the power of the lamp (in watts) (e.g. a 16-W negatoscope by Lamendin et al. [2] and Foti et al. [11]; a 40-W negatoscope by Vilacapoma Guerra [12]) or the type of light bulb (e.g. a LED X-ray viewer by Garizoain et al. [13]). Despite the different light sources used to evaluate root translucency, no in-depth studies have been conducted on the influence of the light source on the evaluation of this parameter. Recently, Adserias-Garriga et al. [14] carried out a study on the light conditions used to acquire the root translucency measurements. They evaluated three diverse types of lights with different luminous intensities, 6500 lx (equivalent to microscopic light), 3000 lx (equivalent to negatoscope light) and 1600 lx (equivalent to daily sunlight), and concluded that lighting should be considered to obtain a reliable estimation of age-at-death.

Together with illuminance level, colour temperature is the other important characteristic of light to be considered regarding human visual perception [15]. However, this characteristic has not been investigated in previous studies. The colour temperature of a light source is defined as the colour of light emitted of an opaque and non-reflective black-body whose colour is closest to that of the light source (i.e. it is a way to describe the light appearance provided by a light source) [16]. By convention, the colour temperature is expressed by the unit of absolute temperature, the Kelvin (K). Light from warm white light sources appear yellow white and has a colour temperature between about 2700 and 3500 K. Cool white light is seen as blue white with colour temperatures ranging from 4500 to 7500 K. Light sources with colour temperatures in the middle range (3500–4500 K) are described as neutral white. Currently, the lighting industry formally refers to warm white (3000 K), neutral white (3500 K), cool white (4000–4500 K) and daylight (6500 K), based on the ANSI standard [16, 17].

Today, there are many LED light tables on the market that are used to evaluate the root translucency (e.g. [13, 18]). Their main advantage is their low cost and versatility due to their light stability and digital control. These LED light tables have a light illuminance level and colour temperature that can be adjusted through LED drivers, allowing digital control of the light emission and mixing of the different light parameters to adapt it to the requirements of different scenarios. Thus, the LED light spectrum will not only influence the chromatic aspect of the emitted light but also the colour perception of the objects illuminated by this light source. The aim of the present study is to analyse the influence of illuminance and colour temperature of a LED light source when the root translucency is evaluated to optimize the use of Lamendin’s technique for age-at-death estimation.

## Materials and methods

### Sample and measurement collection

Fifty-one permanent teeth were clinically extracted at the Department of Medical and Oral Sciences and Biotechnologies of the ‘G. d’Annunzio’ University of Chieti-Pescara (Italy). Teeth were extracted due to periodontal reasons and made available for educational purposes without any identifying information. Teeth were extracted clinically from their sockets, washed with water, digested for 5 min in a 0.05% solution of sodium hypochlorite, dried and placed in plastic bags. Only single-rooted teeth unaffected by restorations and pathological processes (e.g. root caries, internal root resorption) were included in the study. Thus, from the original sample of 51 permanent teeth and after the application of inclusion/exclusion criteria, the final study sample consisted of a total of 30 teeth (6 maxillary and 21 mandibular incisors, 2 mandibular canines, 1 mandibular premolar).

A digital dental caliper (Masel Orthodontics Inc., USA) was used for the collection of measurements to an accuracy of 0.01 mm. The measurements were taken following the technique outlined by Lamendin et al. [2], involving root height (RH), periodontal regression height (PH) and root translucency height (RTH). RH was defined as the maximum distance from the apex of the root to the cemento-enamel junction. PH was defined as the maximum distance from the cemento-enamel junction to the line of soft tissue attachment. RTH was measured as the maximum extent of the translucent zone from the apex of the root. All measurements were taken macroscopically on non-sectioned teeth from the labial surface along the longitudinal axis of the tooth, and all values were recorded in millimetres. All teeth were measured for RH and PH under a LED desk lamp, and then, they were placed on a LED light table (Ohuhu, USA) in a dark room to measure RTH. This LED light table is adjustable, allowing different colour temperatures and illuminance intensities to be selected. According to the specifications of the manufacturer, the three colour temperatures of this LED light table were defined as cool white (5000 K), neutral white (4000 K) and warm white (2700 K). Illuminance intensity was not defined by the manufacturer. To establish the illuminance intensity of the three levels (maximum, medium, minimum) of the LED light table, a KPS-LX30LED luxmeter (KPS, Spain) was used. Ten measurements were collected for each combination of illuminance level and colour temperature, and the mean value was calculated as the reference value for defining the illuminance of this LED light table (Table 1).

**Table 1:** Light settings (colour temperature and illuminance) of the LED light table

Colour temperature	Illuminance (in lux)					
	Level	*N*	Max	Min	Mean	SD
Cool white (5000 K)	Maximum	10	2880	2840	2863	13.17
	Medium	10	1780	1749	1764	9.62
	Minimum	10	119	116	118	0.81
Neutral white (4000 K)	Maximum	10	2000	1961	1979	11.33
	Medium	10	1213	1186	1200	9.82
	Minimum	10	80	77	78	1.01
Warm white (2700 K)	Maximum	10	2230	2190	2210	14.14
	Medium	10	1521	1494	1508	9.08
	Minimum	10	99	97	98	0.59

Abbreviations: *N*, number of teeth; *Max*, maximum value; *Min*, minimum value; *Mean*, mean value; SD, standard deviation

### Examiners and training session in dental measurements

Dental measurements were collected by three examiners with different backgrounds in dental anthropology: (i) the first examiner has a PhD degree and is highly experienced in odontometrics and Lamendin’s technique (experienced examiner); (ii) the second examiner is a PhD student with extensive knowledge in dental morphology and trained in odontometrics but without prior training in Lamendin’s technique (intermediate examiner) and (iii) the third examiner is a PhD student without previous knowledge in dental morphology and no prior training in odontometrics and Lamendin’s technique (inexperienced examiner).

To train the intermediate and inexperienced examiners, a 3-h training session was conducted by the experienced examiner to recognize dental features and to correctly collect the RH, PH and RTH measurements following the original technique of Lamendin et al. [2], as well as for the correct use and adjustment of light settings (colour temperature and illuminance) of the LED light table. For this training procedure, the intermediate and inexperienced examiners measured a sample of 12 teeth in two sessions with direct feedback from the experienced examiner.

### Study design

**Fig. 1:**
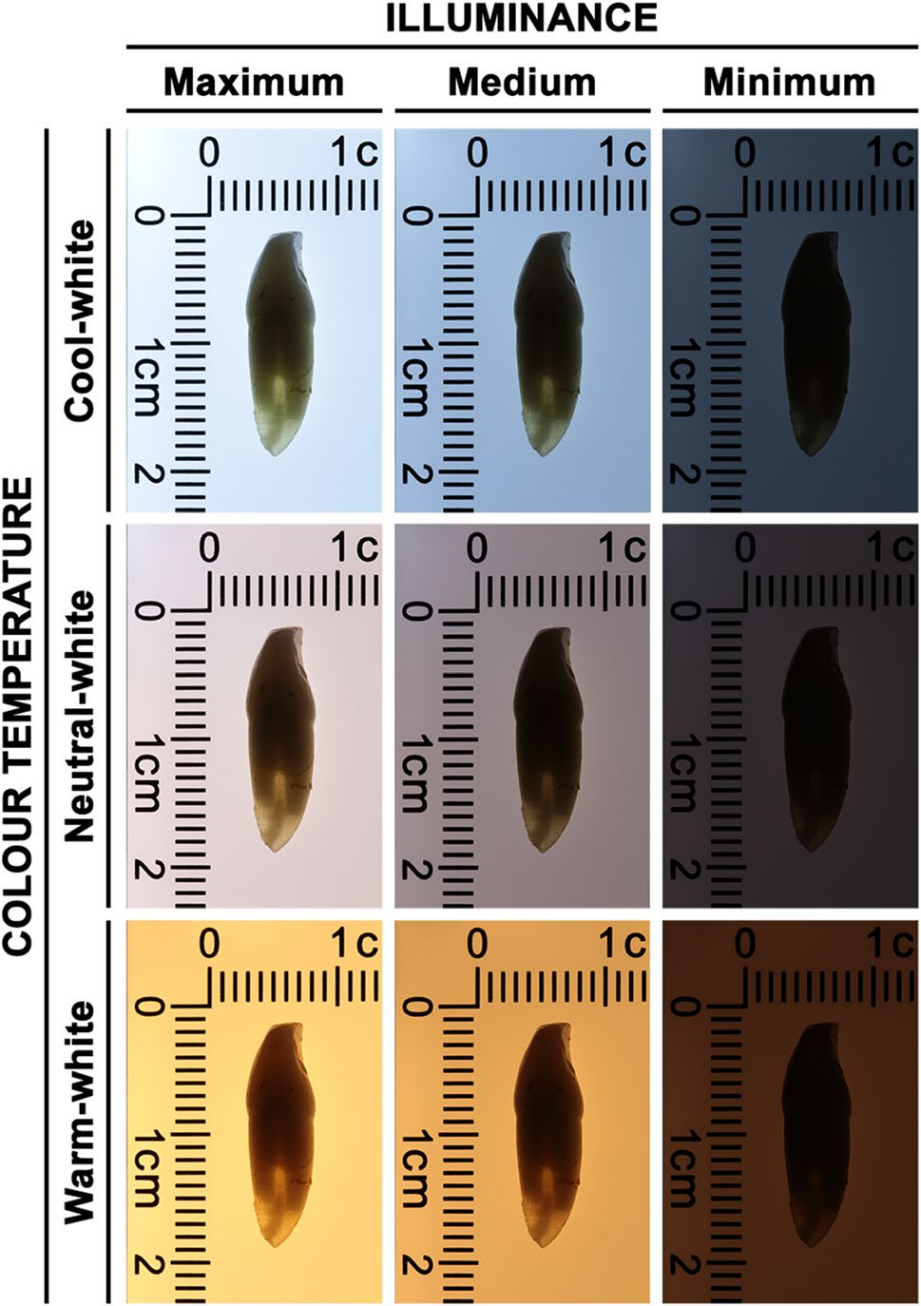
View of root translucency height (RTH) of the same tooth with the different light settings (i.e. different combinations of colour temperature and illuminance) as defined in Table 1

**Fig. 2:**
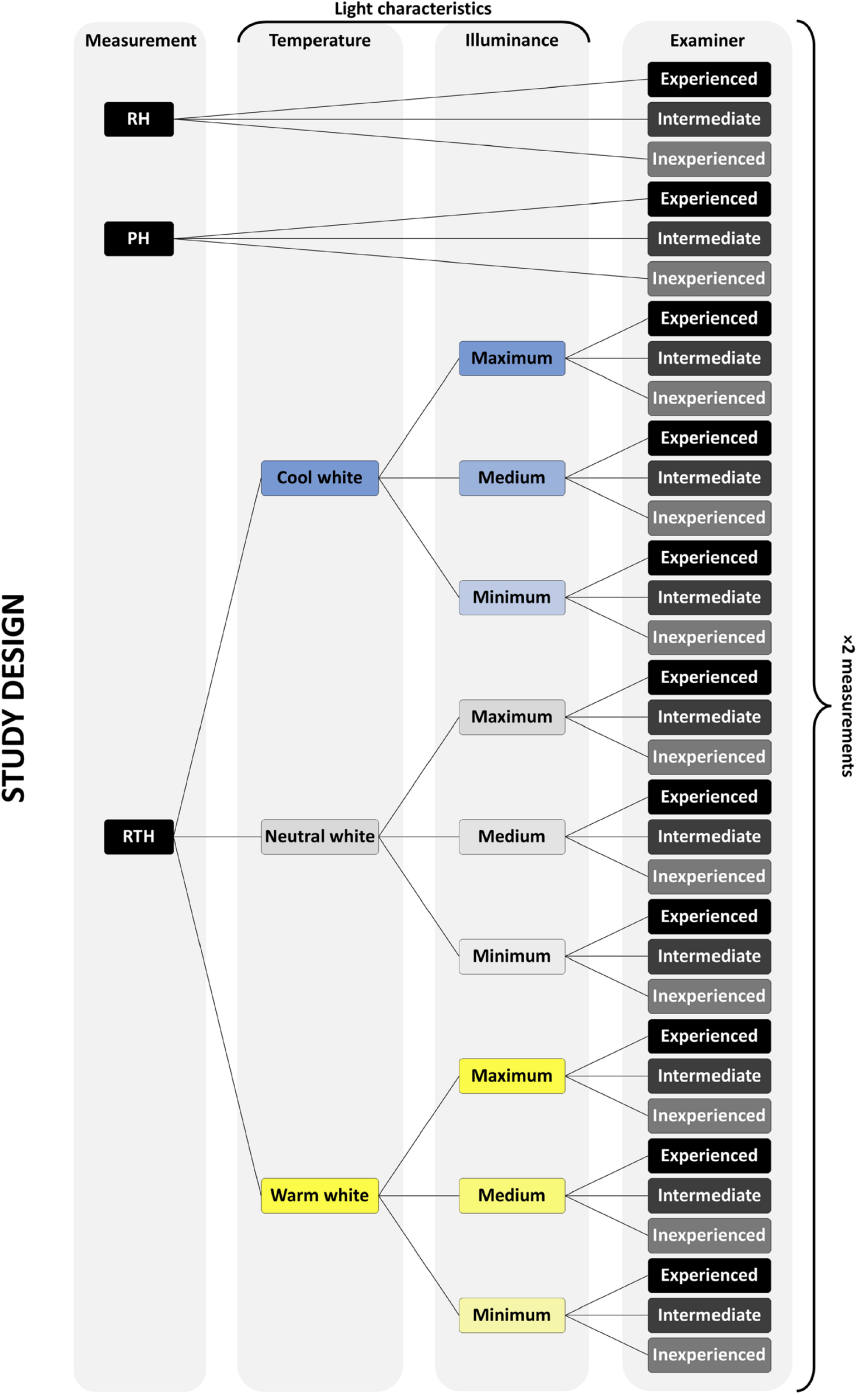
Schematic representation of the data collection procedure for each measurement

All three examiners (experienced, intermediate and inexperienced) collected RH, PH and RTH measurements using the same set of calipers and LED light table. All measurements were repeated at separate times, with a minimum period of 2 weeks and a maximum of 1 month between the two measurements. RTH was measured applying different combinations of colour temperature and illuminance (Fig. 1). Figure 2 illustrates the study design that was followed. All measurements were directly collected into a preformatted Microsoft Excel worksheet. Both randomization and blinding procedures were conducted to guarantee higher-quality data collection by preventing any subjective bias. Randomization ensured that teeth were randomly assigned to different combinations of colour temperature and illuminance for each examiner to prevent systematic arrangement during measurement procedure and to avoid predictability. In addition, the examiner entering the data onto the preformatted worksheet was different from the examiner taking the measurements to prevent selection bias and increase objectivity among the examiners. After taking the measurements, the Prince and Ubelaker formulae [3] to estimate age-at-death were applied. Due to the fact that the Prince and Ubelaker formulae are differentiated by sex and that this biological parameter is unknown in our specimens, in order to apply the age-at-death estimation formulae, sex was randomly assigned using the Excel RAND function.

### Statistical analysis

All statistical analyses were conducted using the statistical package for social sciences software IBM SPSS Statistics 25.0 for Windows [19]. Initially, the differences between the means of the repeated measurements collected at the two separate times by the three examiners were analysed to evaluate possible intraexaminer and interexaminer error. The intraclass correlation coefficient (ICC) was calculated to determine the level of agreement between the repeated measurements collected by the same examiner and by the different examiners. The ICC calculations were conducted using the “two-way mixed-effects absolute-agreement” model for both the intraexaminer and interexaminer errors. As the measure of the intraexaminer and interexaminer agreement, the ICC and its 95% confidence interval (95% CI) were calculated. To determine the degree of agreement, the ICC calculated was compared to the criteria proposed by Koo and Li [20], which establishes four levels of qualitative assessment: ICC > 0.9 indicates “excellent” reliability; ICC from 0.75 to 0.9 indicates “good” reliability; ICC from 0.5 to 0.75 indicates “moderate” reliability and ICC < 0.5 indicates “poor” reliability.

Next, after the verification of the potential intraexaminer and interexaminer error, the average of the repeated measurements collected at the two separate times was used to adjust the values for the subsequent analyses. Then, the data were assessed for normality using the Shapiro–Wilk test, with *p* < 0.05 defining statistical significance. A two-way repeated measures ANOVA was run to determine if the differences in the RTH measurements collected by the different examiners (within subjects) were influenced by the colour temperature and illuminance of the LED light source. Finally, after the application of the Prince and Ubelaker formulae [3] for age-at-death estimation, a three-way ANOVA was run to examine the effect of the examiner’s perception, colour temperature and illuminance on the estimated age-at death*.* For both the two-way and three-way repeated measures ANOVA, Mauchly’s test of sphericity was used to verify if the sample covariance matrix violated the assumptions of the repeated measures ANOVA. If the assumption of sphericity was violated, to avoid the risk of increasing the Type I error, corrections were applied to produce a valid *F*-value. For this purpose, the degrees of freedom for the effect were adjusted (when estimated epsilon [ε] was greater than 0.75, then the Huynh–Feldt correction was used; when ε was less than 0.75, then the Greenhouse–Geisser correction was used). Because multiple pairwise comparisons were performed, the Bonferroni correction was applied to adjust the significance level (*p*-value) to keep the type I error at 5% overall. To interpret the measure of the effect size provided by the partial eta-squared (*η*_*p*_^2^), threshold values of the effect size were interpreted as a small (0.01), medium (0.06) or large effect (> 0.14) [21].

## Results

### Intraexaminer and interexaminer error analyses

In the intraexaminer error analysis (Table 2), the experienced examiner showed similar results for all the measurements (RH, PH and RTH), with ICC values ranging from 0.980 to 0.998. For the intermediate examiner, the ICC values for the RH and PH measurements ranged from 0.976 to 0.988, with slightly lower ICC values of 0.868–0.986 for the RTH measurements. For the inexperienced examiner, the ICC values for the RH and PH measurements were high, ranging from 0.971 to 0.975, with low ICC values of 0.080–0.975 for the RTH measurements.

**Table 2:** Comparison of differences in the means for RH, PH and RTH measurements between repeated measurements within the examiners (i.e. intraexaminer error analysis)

						95% CI				
Examiner	Measurement	Temperature	Illuminance	*N*	ICC	Lower	Upper	*F*	*p*	Strength of agreement
Experienced	RH			30	0.998	0.996	0.999	496.087	0.000	Excellent
	PH			30	0.995	0.988	0.997	176.421	0.000	Excellent
	RTH	Cool white	Maximum	30	0.993	0.986	0.997	145.474	0.000	Excellent
			Medium	30	0.993	0.985	0.997	139.718	0.000	Excellent
			Minimum	30	0.980	0.954	0.991	54.893	0.000	Excellent
		Neutral white	Maximum	30	0.992	0.962	0.997	190.905	0.000	Excellent
			Medium	30	0.996	0.991	0.998	227.142	0.000	Excellent
			Minimum	30	0.981	0.953	0.992	62.075	0.000	Excellent
		Warm white	Maximum	30	0.988	0.976	0.994	83.865	0.000	Excellent
			Medium	30	0.996	0.991	0.998	224.969	0.000	Excellent
			Minimum	30	0.988	0.975	0.994	81.241	0.000	Excellent
Intermediate	RH			30	0.985	0.969	0.993	66.213	0.000	Excellent
	PH			30	0.976	0.949	0.989	44.833	0.000	Excellent
	RTH	Cool white	Maximum	30	0.903	0.781	0.955	11.672	0.000	Excellent
			Medium	30	0.986	0.970	0.993	71.495	0.000	Excellent
			Minimum	30	0.919	0.800	0.964	14.922	0.000	Excellent
		Neutral white	Maximum	30	0.928	0.849	0.966	13.605	0.000	Excellent
			Medium	30	0.971	0.939	0.986	36.363	0.000	Excellent
			Minimum	30	0.893	0.765	0.950	10.331	0.000	Good
		Warm white	Maximum	30	0.901	0.794	0.953	10.160	0.000	Excellent
			Medium	30	0.885	0.668	0.952	11.447	0.000	Good
			Minimum	30	0.868	0.605	0.946	10.186	0.000	Good
Inexperienced	RH			30	0.975	0.913	0.990	56.274	0.000	Excellent
	PH			30	0.971	0.939	0.986	34.394	0.000	Excellent
	RTH	Cool white	Maximum	30	0.973	0.944	0.987	38.195	0.000	Excellent
			Medium	30	0.601	0.166	0.809	2.879	0.003	Moderate
			Minimum	30	0.880	0.749	0.943	8.563	0.000	Good
		Neutral white	Maximum	30	0.862	0.709	0.934	7.047	0.000	Good
			Medium	30	0.791	0.499	0.907	5.829	0.000	Good
			Minimum	30	0.080	− 0.952	0.564	1.086	0.413	Poor
		Warm white	Maximum	30	0.975	0.948	0.988	39.494	0.000	Excellent
			Medium	30	0.770	0.510	0.891	4.809	0.000	Good
			Minimum	30	0.439	− 0.167	0.732	1.787	0.062	Poor

Abbreviations: *RH*, root height; *PH*, periodontal regression height; *RTH*, root translucency height; *N*, number of teeth; *ICC*, intraclass correlation coefficient; *95% CI*, 95% confidence interval; *F*, *F*-statistic; *p*, *p*-value

In the interexaminer error analysis (Table 3), the comparison between the experienced vs. intermediate examiner showed a high agreement for the RH and PH measurements, ranging from 0.981 to 0.984, with slightly lower ICC values of 0.882–0.955 for the RTH measurements. The comparison of the inexperienced examiner with the other two examiners (i.e. experienced vs. inexperienced examiner and intermediate vs. inexperienced examiner) showed similar ICC values. For the RH and PH measurements, the ICC values were 0.973–0.974 for the comparison of experienced vs. inexperienced and 0.982–0.991 for the comparison of intermediate vs. inexperienced, with low values for the RTH measurements, from 0.132 to 0.793 for the comparison of experienced vs. inexperienced and from 0.170 to 0.929 for the comparison of intermediate vs. inexperienced.

**Table 3:** Comparison of differences in the means for RH, PH and RTH measurements between repeated measurements between the examiners (i.e. interexaminer error analysis)

						95% CI				
Examiners	Measurement	Temperature	Illuminance	*N*	ICC	Lower	Upper	*F*	*p*	Strength of agreement
Experienced vs. intermediate	RH			30	0.981	0.883	0.994	89.124	0.000	Excellent
	PH			30	0.984	0.966	0.992	61.202	0.000	Excellent
	RTH	Cool white	Maximum	30	0.939	0.799	0.976	22.804	0.000	Excellent
			Medium	30	0.912	0.805	0.959	12.669	0.000	Excellent
			Minimum	30	0.884	0.666	0.952	11.305	0.000	Good
		Neutral white	Maximum	30	0.955	0.819	0.984	33.612	0.000	Excellent
			Medium	30	0.900	0.672	0.961	14.031	0.000	Excellent
			Minimum	30	0.890	0.747	0.950	10.489	0.000	Good
		Warm white	Maximum	30	0.908	0.608	0.967	17.022	0.000	Excellent
			Medium	30	0.882	0.743	0.945	9.427	0.000	Good
			Minimum	30	0.905	0.715	0.961	14.029	0.000	Excellent
Intermediate vs. inexperienced	RH			30	0.991	0.977	0.996	129.645	0.000	Excellent
	PH			30	0.982	0.961	0.991	55.266	0.000	Excellent
	RTH	Cool white	Maximum	30	0.596	0.145	0.809	2.919	0.003	Moderate
			Medium	30	0.170	− 0.348	0.540	1.305	0.239	Poor
			Minimum	30	0.929	0.835	0.968	16.290	0.000	Excellent
		Neutral white	Maximum	30	0.566	− 0.185	0.829	4.001	0.000	Moderate
			Medium	30	0.211	− 0.381	0.585	1.348	0.213	Poor
			Minimum	30	0.603	0.067	0.823	3.312	0.001	Moderate
		Warm white	Maximum	30	0.590	− 0.136	0.837	4.008	0.000	Moderate
			Medium	30	0.311	− 0.214	0.640	1.687	0.082	Poor
			Minimum	30	0.564	0.034	0.800	2.928	0.003	Moderate
Experienced vs. inexperienced	RH			30	0.974	0.453	0.994	116.428	0.000	Excellent
	PH			30	0.973	0.944	0.987	36.321	0.000	Excellent
	RTH	Cool white	Maximum	30	0.549	− 0.092	0.805	3.222	0.001	Moderate
			Medium	30	0.132	− 0.268	0.478	1.295	0.245	Poor
			Minimum	30	0.793	0.264	0.922	7.530	0.000	Good
		Neutral white	Maximum	30	0.566	− 0.185	0.829	4.001	0.000	Moderate
			Medium	30	0.198	− 0.248	0.543	1.461	0.156	Poor
			Minimum	30	0.482	− 0.178	0.773	2.950	0.002	Poor
		Warm white	Maximum	30	0.590	− 0.136	0.837	4.008	0.000	Moderate
			Medium	30	0.233	− 0.224	0.572	1.614	0.102	Poor
			Minimum	30	0.473	− 0.206	0.773	3.072	0.002	Poor

Abbreviations: *RH*, root height; *PH*, periodontal regression height; *RTH*, root translucency height; *N*, number of teeth; *ICC*, intraclass correlation coefficient; *95% CI*, 95% confidence interval; *F*, *F*-statistic; *p*, *p*-value

The ICC values showed high reproducibility in the intraexaminer error analyses (i.e. with “good” to “excellent” agreements) for both the experienced and intermediate examiners, which indicated that the repeated measurements collected by them were particularly reliable. The overall data for the inexperienced examiner showed lower ICC values (which ranged from “poor” to “excellent” agreement). In addition, the overall data for the inexperienced examiner in comparison with the experienced and intermediate examiner (interexaminer error analyses) showed extremely low ICC values (which ranged from “poor” to “excellent” agreement). For this reason, measurements collected by the inexperienced examiner were excluded from the subsequent analyses and only the data of the experienced and the intermediate examiner were considered for performing the two-way and three-way repeated measures ANOVA analyses.

### Influence of illuminance and colour temperature of a LED light source when tooth translucency is evaluated

The Shapiro–Wilk test showed that the RTH measurements for all combinations of colour temperature and illuminance were normally distributed (*p* > 0.05) for both experienced and intermediate examiners. A factorial ANOVA (two-way repeated measures ANOVA) was conducted to compare the main effects of colour temperature and illuminance as well as their interaction effects on the collection of the RTH measurements, separated by examiners. Table 4 shows the results of the ANOVA for the RTH measurements with different light settings.

**Table 4:** ANOVA for the RTH measurements, depending on the light settings

Examiner	Factor	SS	df	MS	*F*	*p*	*η*_*p*_^*2*^
Experienced	Temperature	0.374	1.692	0.221	2.567	0.095	0.081
	Illuminance	3.324	2	1.662	16.955	0.000	0.369
	Temperature × illuminance	0.619	4	0.155	4.004	0.004	0.121
Intermediate	Temperature	0.522	2	0.261	0.947	0.394	0.032
	Illuminance	3.256	2	1.628	4.089	0.022	0.124
	Temperature × illuminance	3.522	4	0.881	4.874	0.001	0.144

Abbreviations: *SS*, sum of squares; *df*, degrees of freedom; *MS*, mean squares, *F*, *F*-statistic; *p*, *p*-value; *η*_*p*_^*2*^, partial eta square

For the experienced examiner, the main effect of colour temperature was not statistically significant (*p* > 0.05). The main effect of illuminance indicated that 36.9% of the variance on the evaluation of the RTH measurement was explained by the illuminance levels (*F*[2, 58] = 16.955, *p* < 0.001). Multiple comparisons indicated that there were statistically significant differences between the maximum and medium illuminance when compared to the minimum illuminance (*p* < 0.001). Thus, when the experienced examiner measured the RTH, there were statistically significant differences depending on whether this parameter was evaluated with maximum (mean = 5.904, SD = 0.394) or medium (mean = 5.801, SD = 0.384) illuminance in comparison with minimum (mean = 5.634, SD = 0.365) illuminance.

The interaction effect was statistically significant (*F*[4, 116] = 4.004, *p* < 0.01), indicating that there was a combined effect of colour temperature and illuminance level on the evaluation of the RTH measurement, yielding a 12.1% of the variance explained by these combined factors. Multiple comparisons indicated that there were statistically significant differences between the cool-white light and neutral-white light (*p* < 0.05) and between cool-white and warm-white light (*p* < 0.05) when RTH was measured under a minimum illuminance. Thus, when the experienced examiner measured the RTH, if teeth were evaluated with a minimum illuminance, there were statistically significant differences (*p* < 0.05) depending on whether this parameter was evaluated under a cool-white light (mean = 5.515, SD = 0.366), neutral-white light (mean = 5.692, SD = 0.366) or warm-white light (mean = 5.696, SD = 0.369).

Similar results were obtained by the intermediate examiner. The main effect of colour temperature was not statistically significant (*p* > 0.05). The main effect of illuminance indicated that 12.4% of the variance on the evaluation of the RTH measurement was explained by the illuminance levels (*F*[2, 58] = 4.089, *p* < 0.05). Multiple comparisons indicated that there were statistically significant differences between the maximum illuminance when compared to the minimum illuminance (*p* < 0.05). Thus, when the intermediate examiner measured the RTH, there were statistically significant differences depending on whether this parameter was evaluated with maximum (mean = 5.247, SD = 0.393) or minimum (mean = 4.997, SD = 0.362) illuminance.

The interaction effect was statistically significant (*F*[4, 116] = 4.874, *p* = 0.001), indicating that there was a combined effect of colour temperature and illuminance level on the evaluation of the RTH measurement, yielding a 14.4% of the variance explained by these combined factors. Multiple comparisons indicated that there were statistically significant differences between the cool-white light and neutral-white light when RTH was measured under a medium or minimum illuminance (*p* < 0.05). Thus, when the intermediate examiner measured the RTH, if teeth were evaluated with a medium illuminance, there were statistically significant differences (*p* < 0.01) depending on whether this parameter was evaluated under a cool-white light (mean = 5.334, SD = 0.420) or neutral-white light (mean = 5.091, SD = 0.402). When teeth were evaluated under a minimum illuminance, there were also statistically significant differences (*p* < 0.05) in the RTH measurements depending on whether this parameter was evaluated under a cool-white light (mean = 4.821, SD = 0.346) or neutral-white light (mean = 5.150, SD = 0.375).

### Application of the Prince and Ubelaker formulae for age-at-death estimation

Because root translucency is a key parameter for estimating the age-at-death from skeletal remains and in light of the results obtained previously, the Prince and Ubelaker formulae [3] to estimate age-at-death were applied to the sample (see section “Study design” for more details on the sex assignment for the specimens). Thus, a factorial ANOVA (three-way repeated measures ANOVA) was conducted to examine the main effects of examiner, colour temperature and illuminance as well as their interaction effects on the estimation of the age-at-death. The Shapiro–Wilk test showed the data of the estimated age-at-death were normally distributed (*p* > 0.05) for all combinations of colour temperature and illuminance.

The results of the three-way repeated measures ANOVA (Table 5) revealed that the main effect of examiner indicated that 23.2% of the variance on the estimated age-at-death was explained by differences between the examiners when the different measurements (RH, PH and RTH) were collected for the application of the Prince and Ubelaker formulae (*F*[1, 29] = 8.763, *p* < 0.01). A comparison between examiners indicated that there were statistically significant differences (*p* < 0.01) between the experienced (mean = 48.250, SD = 1.236) and the intermediate (mean = 46.675, SD = 1.271) examiner. The main effect of illuminance indicated that 30% of the variance on the evaluation of the estimated age-at-death was explained by the illuminance levels (*F*[2, 58] = 12.424, *p* < 0.001). Multiple comparisons indicated that there were statistically significant differences (*p* < 0.001) between the maximum (mean = 47.764, SD = 1.257) and medium illuminance (mean = 47.590, SD = 1.240) when compared to the minimum illuminance (mean = 47.034, SD = 1.186). No statistically significant main effect on colour temperature was observed (*p* > 0.05).

**Table 5:** ANOVA for the estimated age-at-death after the application of the Prince and Ubelaker formulae [3], depending on examiners and light settings (colour temperature and illuminance) during the collection of the different dental measurements of the sample

Factor	SS	df	MS	*F*	*p*	*η*_*p*_^*2*^
Examiner	335.136	1	335.136	8.763	0.006	0.232
Temperature	7.277	2	3.638	2.672	0.078	0.084
Illuminance	52.340	2	26.170	12.424	0.000	0.300
Examiner × temperature	1.452	2	0.726	0.430	0.653	0.015
Examiner × illuminance	0.812	2	0.406	0.207	0.814	0.007
Temperature × illuminance	23.483	4	5.871	6.574	0.000	0.185
Examiner × temperature × illuminance	11.639	4	2.910	2.931	0.024	0.092

Abbreviations: *SS*, sum of squares; *df*, degrees of freedom; *MS*, mean squares, *F*, *F*-statistic; *p*, *p*-value; *η*_*p*_^*2*^, partial eta square

The interaction effect of temperature × illuminance was statistically significant (*F*[4,116] = 6.574, *p* < 0.001), indicating that there was a combined effect of colour temperature and illuminance level on the estimated age-at-death, yielding an 18.5% of the variance explained by these combined factors. Multiple comparisons indicated that there were statistically significant differences between the cool-white light and neutral-white light (*p* < 0.001) and between cool-white light and warm-white light (*p* < 0.05) when age-at-death was estimated using a minimum illuminance. Thus, under a minimum illuminance of the light source, there were statistically significant differences depending on whether this parameter was evaluated under a cool-white light (mean = 46.589, SD = 1.167), neutral-white light (mean = 47.368, SD = 1.211) or warm-white light (mean = 47.145, SD = 1.193). No statistically significant interaction effect of examiner × temperature and examiner × illuminance was observed (*p* > 0.05).

Finally, the interaction effect of examiner × temperature × illuminance was statistically significant (*F*[4,116] = 2.931, *p* < 0.05), indicating that there was a combined effect of examiner, colour temperature and illuminance level on the estimated age-at-death, yielding a 9.2% of the variance explained by these combined factors. Table 6 shows the multiple comparisons based on the effects of the interaction examiner × temperature × illuminance on the estimated age-at-death. These comparisons indicated statistically significant differences between the experienced and intermediate examiner on the estimated age-at-death in all combinations of colour temperature and illuminance (*p* < 0.01), except the following combinations of colour temperature and illuminance (*p* > 0.05): cool-white light and medium level of illuminance, neutral-white light and minimum level of illuminance and warm-white light and medium level of illuminance.

**Table 6:** Multiple comparisons based on the interaction examiner × temperature × illuminance on the estimated age-at-death

						95% CI	
Examiner comparison	Temperature	Illuminance	Mean differences	SE	*p*	Lower	Upper
Experienced vs. intermediate	Cool white	Maximum	1.568	0.540	0.007	0.464	2.672
		Medium	1.130	0.671	0.103	− 0.242	2.502
		Minimum	1.699	0.616	0.010	0.440	2.958
	Neutral white	Maximum	1.303	0.435	0.006	0.413	2.194
		Medium	2.002	0.618	0.003	0.738	3.266
		Minimum	1.333	0.682	0.060	− 0.061	2.727
	Warm white	Maximum	2.107	0.567	0.001	0.948	3.266
		Medium	1.285	0.697	0.075	− 0.140	2.710
		Minimum	1.752	0.582	0.005	0.561	2.943

Abbreviations: *SE*, standard error; *p*, *p*-value; *95% CI*, 95% confidence interval for difference

## Discussion

From a forensic anthropological perspective, estimation of age-at-death of adult individuals represents a central focus in the identification of human skeletal remains and of the living. Teeth undergo age-related morphological and biochemical changes, including attrition, secondary dentine deposition, gingival regression, cementum apposition, root resorption, dentine translucency and aspartic acid racemization [22, 23]. However, the most frequently considered parameters are secondary dentine deposition (e.g. [24–26]) and root translucency (e.g. [4, 27]), and numerous techniques have been developed to estimate age-at-death from these dental changes.

Lamendin et al. [2] published a simple technique to estimate the age of death in adult individuals, the application of which only requires taking three measurements of a single-rooted tooth: root height (RH), periodontal regression height (PH) and root translucency height (RTH). Thus, this technique and its derivative modifications (e.g. [3, 8]) are based on the use of a caliper to collect the different measurements, as well as a suitable light source to observe and measure the root translucency. However, the collection of these measurements is not free of error, either due to instrument inaccuracy and/or human inconsistencies. In this sense, multiple measurements of the same variable will not always be the same due to variability inherent to the measurement process [28]. These sources of variation can be avoided, or at least minimized, to a large degree. In any metric study, the evaluation and control of the variability of the measurements conducted by the same examiner (repeatability) or different examiners (reproducibility) at separate times are very important because the method calibration and the reliability of the results of the estimated age-at-death will depend on these evaluations [5]. Here, the ICC values showed high repeatability in the intraexaminer error analyses for the RH and PH measurements in the experienced, intermediate and inexperienced examiners (i.e. “excellent” agreements), which indicated that the repeated RH and PH measurements were particularly reliable by the different examiners. However, for the RTH measurements, the ICC values were high for the experienced examiner (i.e. “excellent” agreements), slightly lower for the intermediate examiner (i.e. from “good” to “excellent”) and extremely low for the inexperienced examiner (i.e. from “poor” to “excellent”), indicating a downward trend in reliability in the RTH measurement as the experience of the examiner decreases. For the interexaminer error analyses, the overall data showed high agreement for the RH and PH measurements between examiners (i.e. “excellent” agreements), which indicated that the repeated RH and PH measurements were particularly reliable. However, for the RTH measurements, the ICC values were high between the experienced and intermediate examiner (i.e. from “good” to “excellent”), with extremely low values between the inexperienced examiner and the experienced (i.e. from “poor” to “good”) or intermediate examiner (i.e. from “poor” to “excellent”). The ICC values between the examiners tended to be lower for the RTH than for the RH and PH measurements, which indicated that this particular measurement is more difficult to perform consistently due to the difficulty in finding a definite limit in the transition between the opaque and translucent root dentine. Thus, the evaluation and measurement of RTH are the major source of variability within and between examiners, probably because this measurement is highly dependent on the visual perception of the root translucency and on the subjective judgement of the examiners to delimit the line between the opaque and the translucent dentine.

### Visual perception process of root translucency

Visual perception is defined as the brain’s ability to perceive and interpret the surrounding environment through processing information contained in the visible spectrum of light, reflected by objects in the environment and as influenced by context and experience [16, 29]. There are three components involved in the visual perception process of an object: a light source; an object and a human observer. All three components affect the appearance of the object visualized by the observer [16]. A light source, characterized by the emitted energy at different wavelengths, illuminates the object. When light falls on the object, depending on the physicochemical properties of this object, the light beam is modified by physical processes such as absorption or scattering. Thus, the light reaches the observer’s eye as reflected or refracted light. Light-sensitive pigments in the retina (i.e. cones and rods) absorb light energy, converting the light into electrical impulses that are transmitted via nerve fibres to the brain. The human eye–brain connection makes an instantaneous and continuous assessment of the appearance of the object, and in this way, the light that enters through the eyes includes the distinctive imprints of the light source and the object [16].

**Fig. 3:**
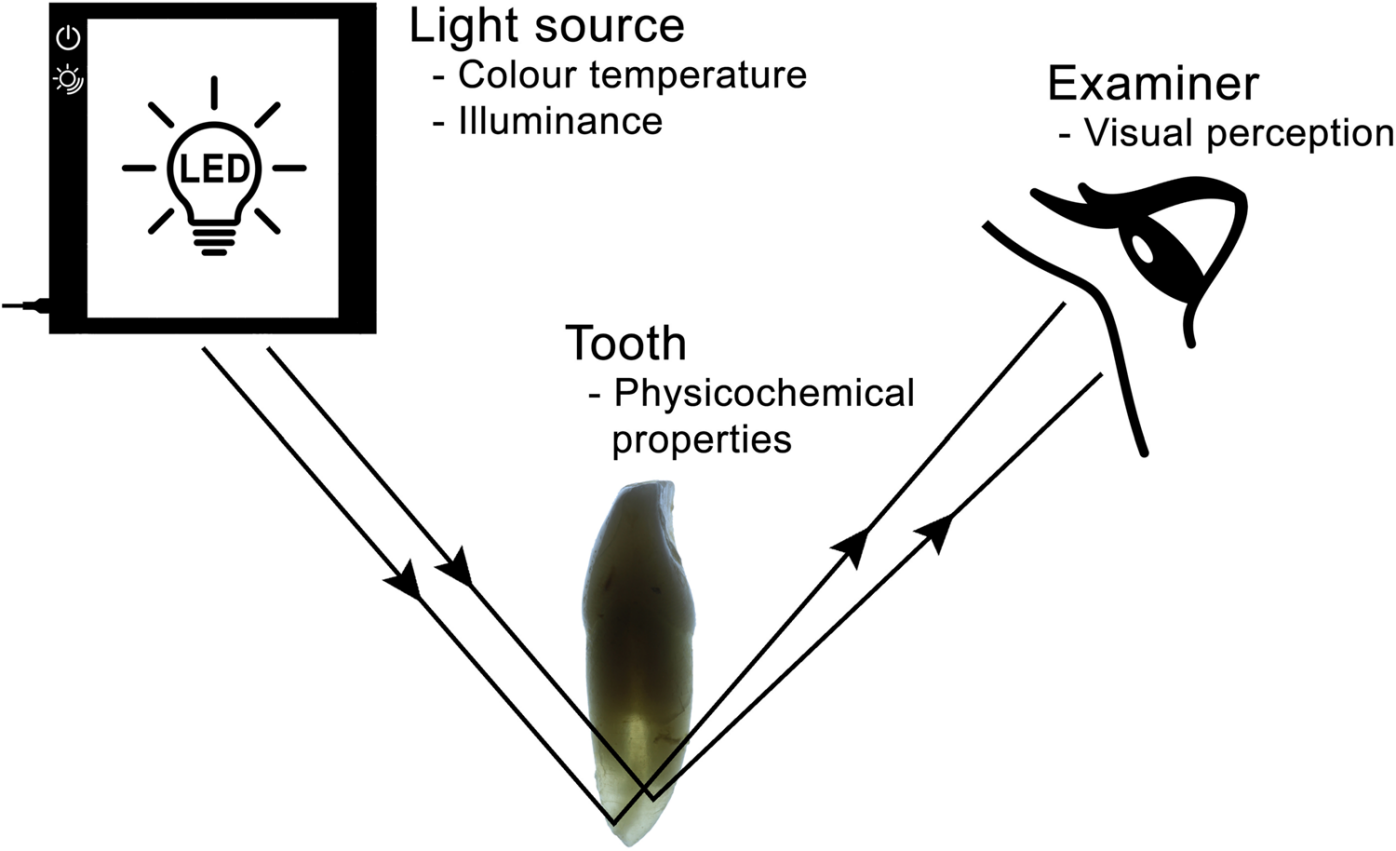
Process of visual perception of the root translucency that is influenced by LED light settings and the physicochemical properties of the tooth

However, when root translucency is evaluated, the light beam passing through the dental root is partially scattered, reflected and refracted. Thus, root translucency can be defined as the physicochemical and structural properties of the dental dentine and cementum that allow the light to pass through them. It is defined as an intermediate situation between opacity (i.e. total blockage of the passage of light) and transparency (i.e. total passage of light). Figure 3 shows that the translucent root allows light to partially pass through it, varying the visual perception of the translucency to a greater or lesser degree according to the physical and chemical properties of the involved dental tissues.

Humans are capable of estimating translucency consistently from objects with different shapes and under different lighting conditions (i.e. exhibiting constancy of translucency). Nevertheless, there can be large constancy failures, with the shape complexity of the object, lighting direction, illuminance level and scattering phase function playing a significant role in the variations in translucent perception [30, 31].

Despite root translucency being a key element in the technique of age-at-death estimation in adults, light settings for measuring this parameter have not been adequately established. Colour temperature and illuminance are the two most important characteristics of light that must be considered regarding human visual perception of translucency [15, 32]. Our study shows that illuminance levels indicate a large (for experienced examiner) or medium (for intermediate examiner) effect on the evaluation of the RTH measurement, showing no differences when the main effect of colour temperature is analysed. Although Adserias-Garriga et al. [14] did not analyse the colour temperature, our results agree with them. Comparing three light sources with different levels of illuminance (6500 lx, 3000 lx and 1600 lx), they concluded that the light illuminance to evaluate the RTH measurement should be 1600 lx because the other higher illuminance levels influence the measurement of root translucency. Our results show that maximum (range: 1961–2880 lx) and medium (range: 1186–1780 lx) illuminance levels do not affect the perception for the RTH measurement, while minimum illuminance levels (range: 76–119 lx) have a significant effect. However, the interaction temperature × illuminance shows significant differences when the different examiners evaluated the root translucency (medium effect by the experienced examiner, large effect by the intermediate examiner); therefore, both factors should be considered for collecting RTH measurements. In addition, when the Prince and Ubelaker formulae [3] to estimate age-at-death were applied to the sample by the experienced and intermediate examiners, the results confirmed the previous observations. The examiners indicated a large effect on the estimation of age-at-death. Although the ICC values were remarkably high between the experienced and intermediate examiners, the large variance on the estimated age-at-death (23.2%) could be explained by the different visual perceptions that different examiners have when evaluating root transparency with different light settings. The results of the interaction examiner × temperature × illuminance confirm this hypothesis because there is a medium effect, so the estimation of age-at-death is affected by these combined factors.

In summary, the evaluation and measurement of RTH is the major source of variability within and between examiners, probably because this measurement is highly dependent on the visual perception of the root translucency (due to the influence of light conditions) and on the subjective judgement of the examiners to delimit the line between the opaque and the translucent dentine. That is, when different examiners delimit the line that serves as a reference to take the RTH measurement, the placement of this landmark may be different as a consequence of the differential visual perception of each of the examiners. In this way, the visual perception of the root translucency of each of the examiners will depend on the differences in the anatomy and physiology of the cone and rod systems of their visual system, which will also be influenced by the physicochemical characteristics of the dental tissues themselves and by the characteristics of the light source. Therefore, differences in the age-at death estimation of Lamendin’s technique will not only depend on the examiner’s experience and the accuracy of the method itself but will also be influenced by the examiner’s own visual perception of root translucency. This situation results in a subjective assessment of the age estimate. Humans perceive light in an absolutely different way than scientific equipment, which means that human perception is intrinsically biased when trying to see colour and light. Thus, various alternative methods have been proposed to evaluate root translucency objectively, mainly using scientific equipment and digital aids (e.g. [33, 34]), with research concluding that computer-based translucency measurements contributed best to the age-at-death estimation in comparison to conventional and subjective caliper-based methods (e.g. [4, 35–38]). However, all these methods are based on sectioned teeth and not intact teeth. In the case of intact teeth, it is essential to highlight the transilluminator developed by Olze et al. [39] for measuring root dentine translucency in a predetermined, standard manner. However, due to the lack of commercial distribution of devices specially designed for this purpose and to optimize the use of the age-at-death estimation technique, the evaluation and measurement of root translucency should be recorded under the same light conditions as the reference study (i.e. according to the specific-population method developed to estimate age-at-death of unidentified bodies).

## Conclusion

The aim of this study was not to establish the specific parameters of light conditions to acquire the root translucency measurement but rather to conduct an exploratory analysis of the influence of illuminance and colour temperature of the light source on the evaluation of root translucency to optimize the use of Lamendin’s technique for age-at-death estimation. This study describes how light conditions (colour temperature and illuminance) may affect different examiner’s visual perception of root translucency and, therefore, affect age-at-death estimation methods and techniques based on this parameter. Although this study represents an important contribution to the topic of the influence of light source on the evaluation of root translucency, there are some limitations that should be considered. In this study, we only analysed three specific colour temperatures and three levels of illuminance intensity; therefore, future research should include a wider range of these parameters to determine which combination of colour temperature and illuminance are best for evaluating root translucency and correlates better with age-at-death. In addition, future research should also include a large selection of known-aged and sexed dental specimens and diverse classes of maxillary and mandibular single-rooted teeth. Thus, further studies are required to expand these results to establish the correct and standard light settings necessary to optimize the age-at-death estimation using intact teeth, and the exploration of alternatives using more objective methods based on digital aids is recommended to circumvent the influence of human visual perception of root translucency.

## Data Availability

The datasets generated during and/or analysed during the current study are available from the corresponding author on reasonable request.
